# Corrosion resistance of coupled sandblasted, large‐grit, acid‐etched (SLA) and anodized Ti implant surfaces in synthetic saliva

**DOI:** 10.1002/cre2.198

**Published:** 2019-07-25

**Authors:** Ala'a Al Otaibi, El‐Sayed M. Sherif, Mohammed Q. Al‐Rifaiy, Spiros Zinelis, Youssef S. Al Jabbari

**Affiliations:** ^1^ Dental Biomaterials Research and Development Chair, College of Dentistry King Saud University Riyadh Saudi Arabia; ^2^ Department of Prosthetic Dental Science, College of Dentistry King Saud University Riyadh Saudi Arabia; ^3^ Center of Excellence for Research in Engineering Materials (CEREM) King Saud University Riyadh Saudi Arabia; ^4^ Electrochemistry and Corrosion Laboratory, Department of Physical Chemistry National Research Centre (NRC) Cairo Egypt; ^5^ Department of Biomaterials, School of Dentistry National and Kapodistrian University of Athens Athens Greece

**Keywords:** dental implant systems, EIS, electrochemical testing, NobelReplace®, Straumann®

## Abstract

**Purpose:**

The purpose of this study was to investigate the corrosion resistance of galvanically coupled SLA and anodized implant surfaces with a Co‐Cr alloy.

**Materials and Methods:**

Three groups were included in this study. The first (SLA) was composed of SLA implants (Institut Straumann, Basel, Switzerland), the second (ANO) of NobelReplace® (Nobel Biocare, Göteborg, Sweden), and the third (MIX) of both implant systems combined. All groups were assembled with a single Co‐Cr superstructure. Electrochemical testing included open‐circuit potential, electrochemical impedance spectroscopy, cyclic potentiodynamic polarization, and chronoamperometric current‐time measurements. The quantitative results (EOCP, ECORR, ICORR, EPROT, RP, and ICA) were statistically analyzed by one‐way ANOVA and Tukey's post‐hoc multiple comparison test (α = 0.05)

**Results:**

All the aforementioned parameters showed statistically significant differences apart from ECORR and EPROT. The evaluation of qualitative and quantitative results showed that although SLA had higher corrosion resistance compared with ANO, it had less resistance to pitting corrosion. This means that SLA showed increased resistance to uniform corrosion but less resistance if pitting corrosion was initiated. In all cases, MIX showed intermediate behavior.

**Conclusion:**

The corrosion resistance of implant‐retained superstructures is dependent on the electrochemical properties of the implants involved, and thus different degrees of intraoral corrosion resistance among different implant systems are anticipated.

## 1 INTRODUCTION

Contemporary dental implants are made by the use of commercially pure titanium (Ti) and its alloys (mainly Ti‐6Al‐4V), thanks to their osseo‐inductive properties, biocompatibility, corrosion resistance, and good mechanical properties (Le Guehennec, Soueidan, Layrolle, & Amouriq, 2007). The electrochemical properties of medical and dental implants have been experimentally tested in simulated physiological conditions (Barão et al., 2011; Mareci, Chelariu, Gordin, Ungureanu, & Gloriant, 2009; Messer et al., 2010a; Oliveira & Guastaldi, 2009) and have been attributed to the formation of a stable, well‐adhering, and tenacious oxide film (Huang, 2003; Kirmanidou et al., 2016).

The combination of high corrosion resistance with the fact that, in clinical conditions, the implant surface will be covered by soft tissue at the cervical region and by attached bone at the treated Ti surface did not support corrosion as a primary concern. Besides, Ti has shown excellent electrochemical properties in almost neutral solutions (Barão et al., 2012) within the pH range of human saliva (pH 6–7; Dodds, Johnson, & Yeh, 2005). However, oral conditions can change instantly due to temperature and oxygen level fluctuations, even, in a worst‐case scenario, being permanently changed due to peri‐implantitis (Yu, Addison, Baker, & Davenport, 2015), decreasing the local pH and compromising the corrosion resistance of Ti (Souza et al., 2013). Beyond the establishment of a more aggressive environment, peri‐implantitis, followed by the resorption of soft and hard tissues, initially exposing the collar–abutment interface and later the root portion of the implant in the oral environment, can simultaneously trigger two corrosion mechanisms. The first is galvanic coupling among the abutment (either Ti or Ti‐6Al‐4V or a Co–Cr alloy in the case of implant‐retained superstructures), the polished cervical collar, and the treated implant surface. The second is the exposure of a crevice between the implant and the abutment, providing access for oral fluids, further facilitating the decrease in pH and thus leading to corrosion phenomena (Apaza‐Bedoya et al., 2017).

The corrosion of dental implants and other dental alloys is becoming a serious problem, and several studies have been conducted on the effects of corrosion on dental implants and surrounding tissue. It has been reported (Olmedo et al., 2012; Olmedo, Nalli, Verdu, Paparella, & Cabrini, 2013) that corrosion might be implicated in implant failure. Other studies (Aksaka, Yildirim, & Gul, 2004; Hansen, 2008) have stated that mechanical complications, including primarily fatigue fracture, are accelerated by corrosion. Also, implant corrosion can lead to biological complications caused by metal ion release, including toxicity, carcinogenicity, and hypersensitivity (Goodman, Lind, Song, & Smith, 1998). Metallic ions activate macrophages, neutrophils, and T‐lymphocytes and provoke enhanced output of cytokines and metallic proteases (Haynes, Rogers, Hay, Pearcy, & Howie, 1993; Kumazawa et al., 2002). Saini, Singh, Arora, Arora, and Jain (2015) reported that corrosion may also be related to implant failure associated with ionic release and surface roughening, resulting in the discoloration and allergic reactions of adjacent tissues.

Recently, the intraoral corrosion of Ti implants has been described as being among the triggering factors for peri‐implantitis (Mouhyi, Dohan Ehrenfest, & Albrektsson, 2012), whereas a recent study reported increased levels of dissolved Ti in the submucosal plaque from patients with peri‐implantitis compared with that from healthy individuals (Safioti, Kotsakis, Pozhitkov, Chung, & Daubert, 2017). In another study, where bone and mucosal biopsies were tested, a higher amount of Ti was found in 75% of the samples from patients with peri‐implantitis (Fretwurst et al., 2016).

Traditionally, a single dental implant system is used for the treatment and rehabilitation of dental patients. However, in certain situations, a clinician may be confronted with the use of a metallic superstructure connected to different implant systems, with significant differences in the electrochemical properties of treated surfaces (Al Jabbari, Mueller, Al‐Rasheed, & Zinelis, 2016). In such an assembly, the galvanic actions among the different alloys might have a crucial effect on the corrosion resistance of the structure, altering the electrochemical properties of the different components—a scenario that has not been thoroughly investigated.

The aim of this in vitro study was to evaluate the corrosion behavior of two commonly used Ti implant systems, retained with a single Co–Cr superstructure. The null hypothesis was that no significant differences would be identified in the electrochemical properties of the different implant systems.

## 2 MATERIALS AND METHODS

### 2.1 Specimen preparation

Two different implant systems were investigated. The first was the Straumann® system (Institut Straumann, Basel, Switzerland), featuring implants with 4.1‐mm diameter, 12‐mm length, and sandblasted, large‐grit, acid‐etched (SLA) surface treatment. The second system was NobelReplace® (Nobel Biocare, Göteborg, Sweden), with implants 3.5 mm in diameter and 10 mm in length. The surfaces of implants in the second system were treated with TiUnite®, which is characterized by phosphate‐enriched Ti oxide with open craters at the micrometer level (Le Guehennec et al., 2007).

Twelve resin blocks (height, 20 mm; width, 30 mm; and thickness, 10 mm) were divided equally among the three groups, SLA, ANO, and MIX. A 2‐mm drill was placed in a milling machine (Ferraro Engineering, Hereford, AZ, USA) and was used to open two holes about 2‐mm deep. The distance between holes was set at 10 mm, and they were made to indicate the implant positions. Two implants were inserted into each resin block. Two SLA Straumann implants were placed in the SLA group, two NobelReplace implants in the ANO group, and one SLA Straumann and one NobelReplace in the MIX group. The implants were inserted at the preindicated positions into the resin blocks at 50 rpm (Motor unit, Nobel Biocare USA, Yorba Linda, CA, USA), leaving only 2 mm of the treated root region exposed below the collar (about three exposed threads).

The implants of all groups were coupled with a framework made with a Co–Cr dental alloy according to the following procedure. A synOcta abutment (RN synOcta 1.5 screw‐retained abutment, height 1.5 mm) screwed to the Straumann implant fixture and a burnout plastic coping (RN synOcta plastic coping for the synOcta 1.5 bar, height 10.0 mm) were snapped onto the synOcta abutment. A nonengaging burnout plastic coping was snapped onto the Nobel Biocare implant fixture. A plastic bar pattern was positioned between the two plastic cylinders and luted with pattern resin material (Pattern Resin, GC America Inc., Chicago, IL, USA). The specimens were sprued, invested in a gypsum‐bonded investment (Prestobalite, Whip Mix Corp., Louisville, KY, USA), and cast with a Co–Cr alloy (Wirobond 280, Bego, Bremen, Germany; wt%: Co, 60.2; Cr, 25; W, 6.2; Mo, 4.8; Ga, 2.9; Si < 1; Mn < 1) according to the manufacturer's instructions. The cast frameworks were polished and then screwed to the implants by means of Ti SCS occlusal screws (Institut Straumann) for Straumann fixtures and abutment screws (Nobel Biocare AB, Göteborg, Sweden) for Nobel Biocare fixtures. The occlusal screws were placed with the torque recommended by the respective manufacturers.

### 2.2 Synthetic saliva preparation

The artificial saliva solution used was prepared according to ISO10271 (2011) for the electrochemical testing of dental metallic materials, and the chemical composition was as follows (g/dm^3^): Urea, 1.5; NaHCO_3_, 1.5; KCl, 1.2; NaCl, 0.7; KSCN, 0.33; Na_2_HPO_4_, 0.26; and K_2_HPO_4_, 0.2. The pH was calculated by means of a pH meter and adjusted to 6.7 by the addition of drops of lactic acid. The pH meter was calibrated prior to testing by the use of standard solutions of known pH. All experiments were carried out at 37°C with the use of only freshly made solution.

### 2.3 Electrochemical testing

A three‐electrode electrochemical cell was used with Ag/AgCl as a reference electrode, platinum foil as a counter electrode, and the samples as the working electrodes. The electrochemical cell was connected with a potentiostat (Autolab, Metrohm, Utrecht, Netherlands), and all experiments were run in triplicate to ensure the reproducibility of the test. The mean value was used to characterize the sample itself.

### 2.4 Open‐circuit potential

Open‐circuit potential (OCP) curves were recorded immediately after the immersion of electrodes in solution up to the stabilization of measured potential. The final recording after 4,500 s was taken as the E_OPC_ value.

### 2.5 Electrochemical impedance spectroscopy

Nontraditional electrochemical impedance spectroscopy (EIS) experiments were carried out to characterize the electrochemical double‐layer formation and provide further data on corrosion kinetics. The electrochemical process of the given system could then be simulated by an equivalent electrical circuit combining electrical elements with ohmic resistance and capacitance. This equivalent circuit was used for the quantification of the electrochemical processes. EIS data were recorded after 1.0 hr at an OCP value and over a frequency range of 100,000 to 0.1 Hz, with an ac wave of ±5 mV peak‐to‐peak overlaid on a dc bias potential. The EIS data were acquired at a rate of 10 points per decade change in frequency.

### 2.6 Cyclic potentiodynamic polarization

Cyclic potentiodynamic polarization (CPP) experiments were acquired after 1.0 hr of immersion in artificial saliva and performed through scanning of the potential from −800 up to 1,000 mV with a scanning rate of 3.0 mV/s. The protection potential (E_PROT_; where the protective oxide was damaged) and the polarization resistance (R_P_) were measured by CPP curves. Tafel simulation was also used to determine the corrosion potential (E_CORR_) and the corrosion current (I_CORR_).

### 2.7 Chronoamperometry

The samples were initially immersed in artificial saliva for 1 hr, after which a standard potential of 500 mV was applied and the current was constantly recorded over time for 4,500 s (1.25 hr). The final value recorded after 4,500 s was taken as the I_CA_ value.

### 2.8 Statistical analysis

E_OCP_, E_CORR_, I_CORR_, E_PROT_, R_P_, and I_CA_ were statistically analyzed with SPSS (ver. 21) statistical software. Descriptive statistics (mean and standard deviation) were used to describe the aforementioned variables, and one‐way analysis of variance was used to compare the mean values with the three groups as independent variables. Tukey was used as the post hoc multiple comparison test to allocate significant differences among groups. A *p* value of .05 was used to report the statistical significance of results.

## 3 RESULTS

### 3.1 OCP measurements

**Figure 1:**
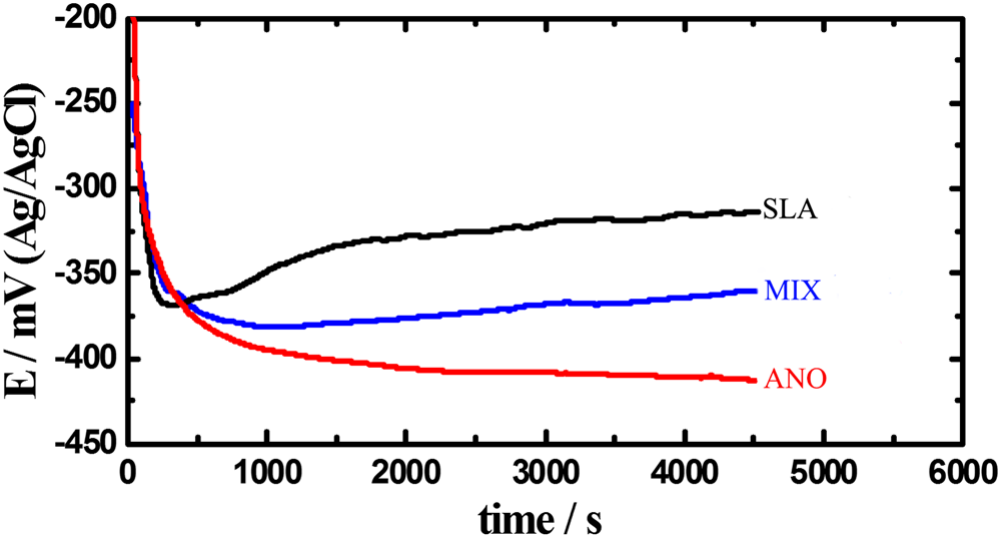
Representative open‐circuit potential curves for all groups tested

The OCP of all groups abruptly shifted toward the negative direction after immersion for the first period of approximately 300 s (Figure 1). The SLA group showed a more stable potential, and after the first 900 s, a slightly more positive potential was maintained until the end of monitoring time. The potential shift to a more noble value was smaller for the MIX group, whereas the shift was toward the negative direction throughout the immersion time for the ANO group, and the stabilization of potential was established after 2,300 s. The average values of E_OCP_ corresponding to 4,500 s (1.25 hr) of immersion were as follows: SLA, −325 ± 12 mV; ANO, −420 ± 20 mV; and MIX, −357 ± 5 mV, with statistically significant differences among them.

### 3.2 EIS measurements

**Figure 2:**
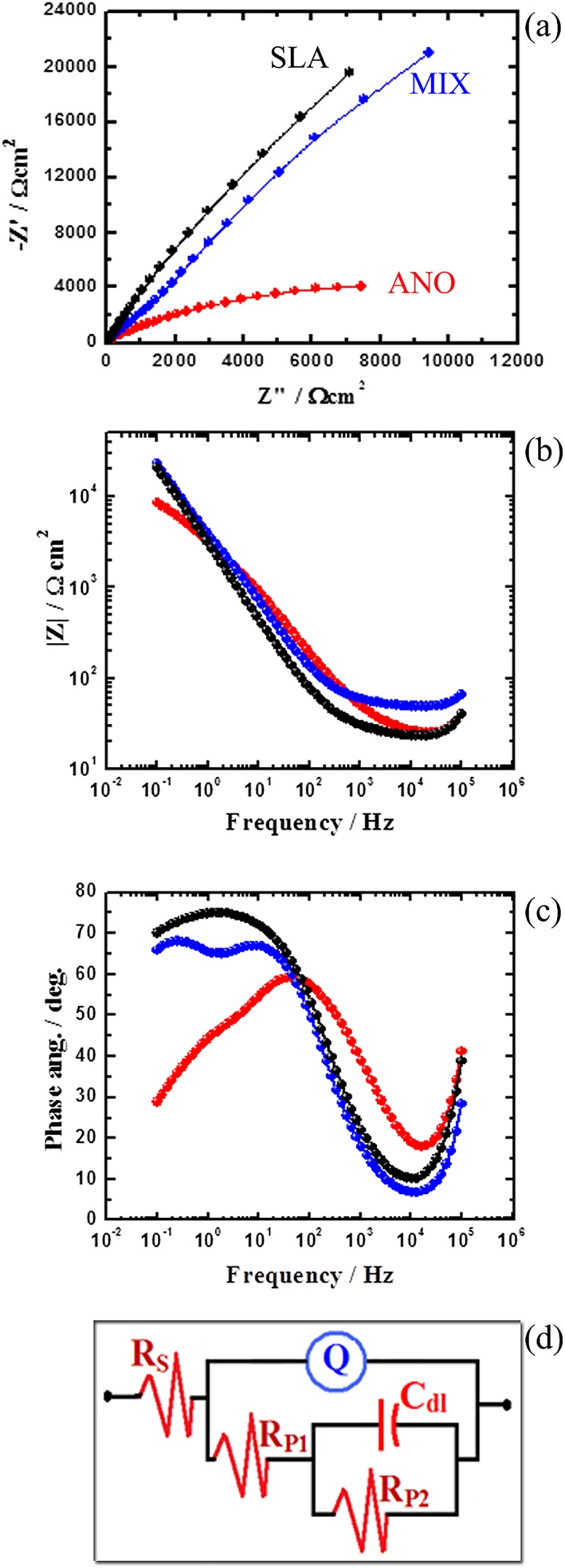
Representative Nyquist (a) plots and Bode plots for impedance (b) and phase angle (c) for SLA, ANO, and MIX. (d) The equivalent circuit after the EIS data were fitted. EIS, electrochemical impedance spectroscopy

The EIS spectra are shown in Figure 2. Nyquist plots (the relation between the real resistance [Z′] and imaginary resistance [Z″]) are shown in Figure 2a, which illustrates a decreased semicircular diameter of the capacitance loop in the ANO group, indicating inferior corrosion resistance compared with the other groups. Bode plots (the absolute values of the impedance [|Z|] and phase angle [Ф] vs. the frequency) were also plotted (Figure 2b,c). It can be seen in Figure 2b that the highest values of |Z|, particularly at the low‐frequency region, were obtained for the SLA and MIX groups. The change in the Bode phase angle with frequency for the three groups is also depicted in Figure 2c, showing that the maximum value of Ф was highest for SLA, followed by MIX and then by ANO.

The EIS spectra were best fitted to the equivalent circuit (Figure 2d), which consists of a duplex oxide layer with an inner barrier film with film resistance R_P2_ and film capacitance C_dl_, whereas the constant phase capacitance element Q stands for the porous outer layer. Y_Q_ and n are the characteristic parameters for the impedance of each element in the circuit, whereas R_P1_ and Rs stand for the polarization resistance inside the pores and solution resistance, respectively. Given that the model is fitted by duplex oxide film layers, the external porous layer is much thicker than the internal one, and thus, the resistance of the pore wall would be so high that its contribution to the circuit may be omitted. Table 1 summarizes the values of all these impedance parameters.

**Table 1:** Electrochemical impedance spectroscopy parameters for the groups tested

Group	EIS parameter					
	R_S_Ω/cm^2^	Q		R_P1_Ω/cm^2^	C_dl_μF/cm^−2^	R_P2_Ω/cm^2^
		Y_Q1_μF/cm^−2^	n_1_			
SLA	56	0.6526	0.83	5,460	2.827	8,296
ANO	42	0.9282	0.80	2,189	3.324	3,734
MIX	51	0.6055	0.73	5,229	2.529	6,911

### 3.3 CPP data

**Figure 3:**
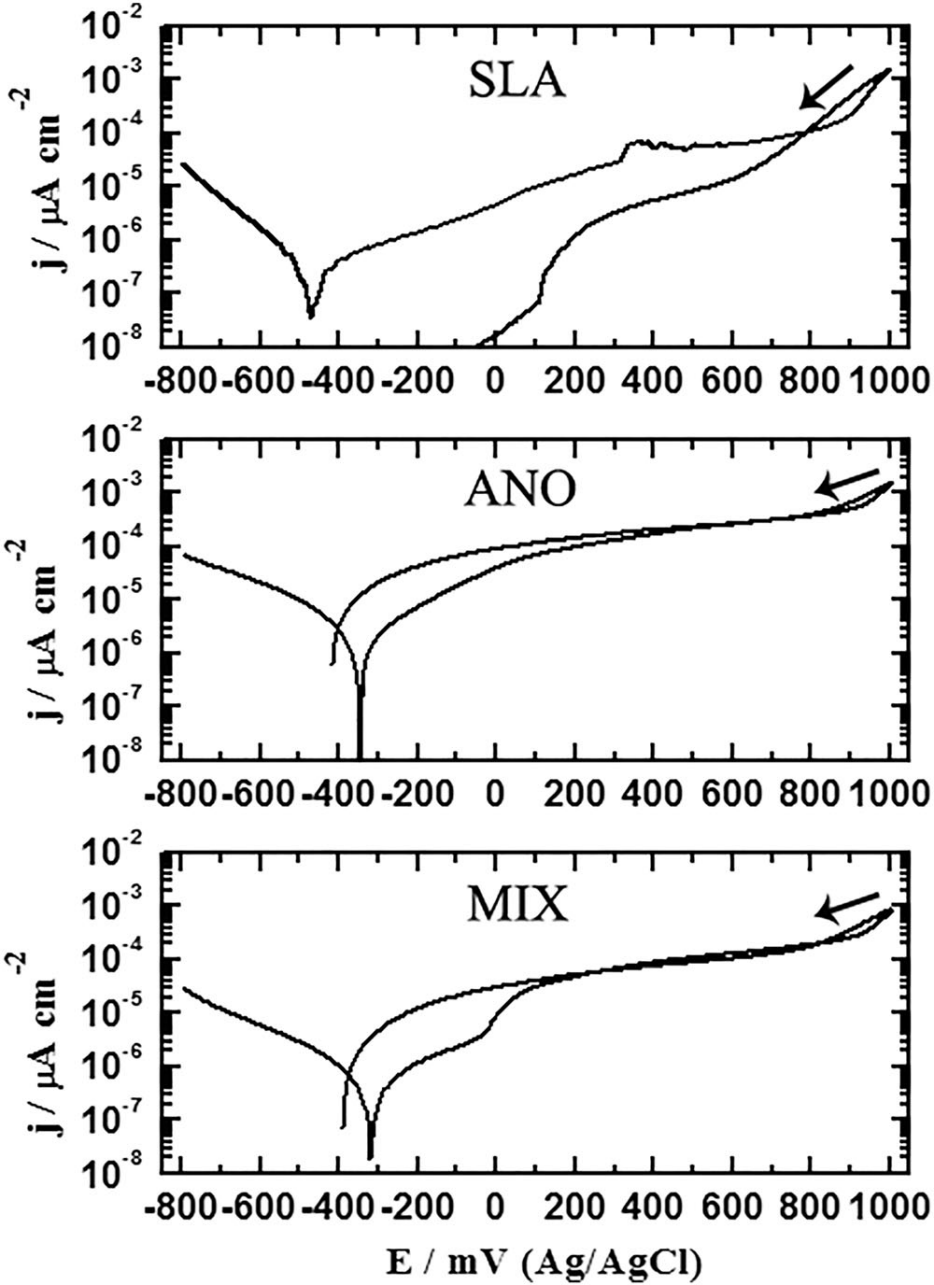
Cyclic potentiodynamic polarization curves recorded for SLA, ANO, and MIX after their immersion in testing solution for 1.0 hr

The CPP curves obtained for SLA, ANO, and MIX are shown in Figure 3. The cathodic potential is associated with the reduction of oxygen, whereas the anodic reaction is associated with the dissolution of metallic structures and the release of electrons, which are consumed in the cathodic reaction (Grosgogeat, Reclaru, Lissac, & Dalard, 1999; Khalil, Sherif, & Almajid, 2011). Similar CPP curves for ANO and MIX were also recorded but with smaller hysteresis loops (the area that arises from the intersection of the backward current and the forward current in the anodic direction on the CPP curve) compared with that obtained for SLA. The CPP behavior for all groups confirmed that the severity of pitting corrosion for SLA was higher than that recorded for the ANO and MIX groups. All groups shared equal E_CORR_ and E_PROT_, but the lowest I_CORR_ was found for SLA, whereas the highest was found for ANO. SLA also showed the highest R_P_ followed by MIX and ANO. Results and statistical outcomes are presented in Table 2.

**Table 2:** Mean values of the outcome variables (E_CORR_, I_CORR_, E_PROT_, and R_P_) obtained for SLA, ANO, and MIX, respectively

Group	E_CORR_ mV	I_CORR_ μA/cm^2^	E_PROT_ mV	R_P_ kΩ/cm^2^
SLA	−454 (13)^a^	0.32 (0.04)^a^	818 (11)^a^	12.7 (2.0)^a^
ANO	−448 (87)^a^	2.55 (0.68)^b^	802 (23)^a^	1.6 (0.8)^b^
MIX	−411 (65)^a^	1.00 (0.50)^c^	820 (14)^a^	5.1 (3.0)^c^

*Note*. Same superscripts denote mean values without statistically significant differences (*p* > .05).

### 3.4 Chronoamperometric current‐time experiments

**Figure 4:**
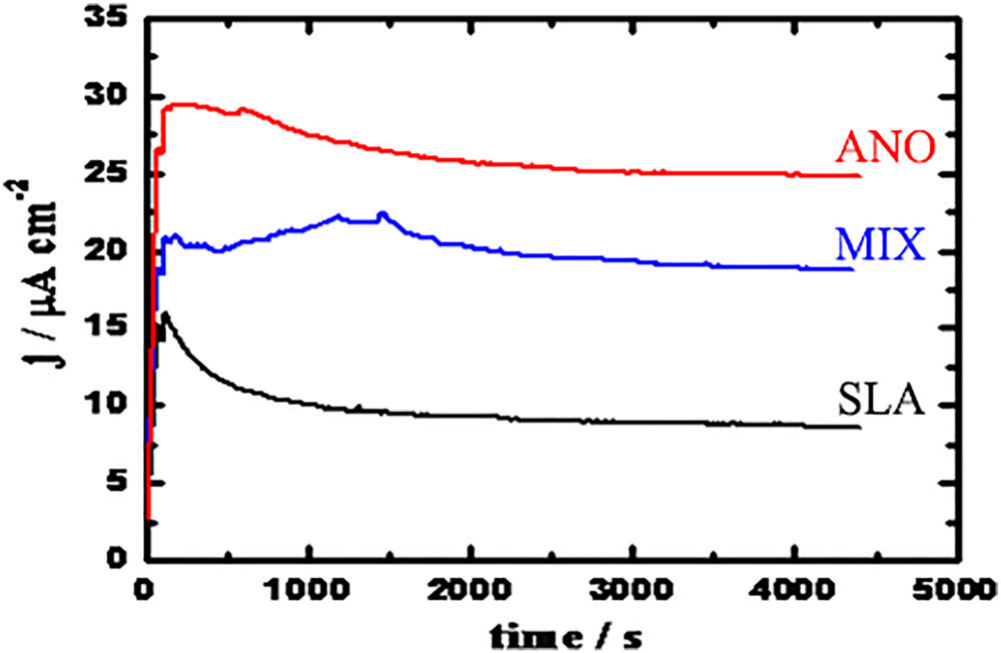
Chronoamperometric current‐time curves obtained for SLA, ANO, and MIX

Figure 4 demonstrates the CCT curves acquired for SLA, ANO, and MIX after their immersion for 1.0 hr in the solution before the application of a constant potential of 0.5 V (Ag/AgCl). It can be noted from Figure 3 that the current values for all tested groups increased in the first few seconds of stepping up of the constant potential. Increasing the time of the experiment for SLA led to decreasing the current values before they steadied by the end of the run. The I_CA_ values were found as follows in ascending order (μα/cm^2^): SLA, 8.9 ± 1.4; MIX, 19.2 ± 2.6; and ANO, 25.3 ± 2.9, with statistically significant differences among them.

## 4 DISCUSSION

According to the results of this study, the electrochemical properties of the three groups were significantly different and thus the null hypothesis must be rejected. SLA (Straumann) and TiUnite (Nobel Biocare) were deliberately selected, because SLA and anodization surface‐roughening techniques had shown maximum differences in their electrochemical properties (Al Jabbari et al., 2016). Therefore, the coupling of these two implants should be considered, from the corrosion standpoint, as the worst‐case scenario.

OCP is the most common and simplest methodology for studying the film formation and passivation of metallic structures. If OCP shifts anodically, the formation of passive film is indicated, whereas a constant OCP value implies that the film remains intact and protective, whereas when OCP moves cathodically, either dissolution of the film or lack of film formation is assumed (Gurappa, 2002). The negative shift of the OCP suggests dissolution of the surface film that had most probably formed on the surfaces of the framework until the formation of a new oxide layer, providing a stable potential during the balance of the immersion time. The alterations of OCP over time for the three groups tested imply that the oxide layer for ANO has less stability than that formed when SLA (Straumann AG) was connected to TiUnite (Nobel Biocare AB) or when two SLA (Straumann AG) implants were connected, which showed the best stability of the oxide film formed. These differences in oxide layer stability among the groups may be explained by the galvanic coupling of treated Ti surfaces and Co–Cr alloy. Although anodization provides maximum corrosion resistance for Ti surfaces (Al Jabbari et al., 2016; Kim, Lee, Piao, Chung, & Kim, 2013; Messer et al., 2010b; Singh et al., 2013; Song, Kim, Jung, Vang, & Park, 2007), this might strengthen galvanic action to the machined collar region and Co–Cr alloy, decreasing their corrosion resistance (Oh & Kim, 2004).

In EIS spectra, the increased diameter of the obtained semicircle indicates a higher passivation of the surface film (AlOtaibi, Sherif, Zinelis, & Al Jabbari, 2016; Khalil et al., 2011). Accordingly, the diameter of the semicircle given by the Nyquist plot classifies the tested groups in the following decreasing order: SLA > MIX > ANO. Table 1 also shows that the listed values of all resistances (R_S_, R_P1_, and R_P2_) recorded the highest values for SLA, but the lowest were always for ANO. The values of Q (CPEs) with their *n* values close to unity are associated with the development of a double‐layer capacitor with the outer layer formed on the surface of the implant fixture for both systems among the three groups, containing some pores where corrosion reactions developed (AlOtaibi et al., 2016; Khalil et al., 2011).

The nature of all anodic polarization curves indicates a low free corrosion activity and a stable anodic and transpassive behavior, in accordance with results from a previous study where Ti implants were connected to a Co–Cr superstructure (Lorenzetti et al., 2014). It was also noted, in reverse scan, that the current was increased, a finding that might be attributed to pitting corrosion. This was represented by the formation of a hysteresis loop. The bigger the size of the area of the hysteresis loop, the more severe the pitting corrosion. The CPP behavior for all groups confirmed that the severity of pitting corrosion for SLA was higher than that recorded for ANO and MIX implant systems. The appearance of pitting corrosion in the SLA group may be attributed to surface roughening, because this has been found previously for SLA surfaces after electrochemical testing (Al Jabbari et al., 2016). The pitting corrosion on the implant Ti surface has been observed in fluorinated solutions, as reported by Cheng et al. (2013). In another study, the examination of five failed Ti implants due to peri‐implantitis provided evidence that Ti dental implants are highly susceptible to pitting attack in the oral environment (Rodrigues et al., 2013). It is generally accepted that increased I_CORR_ increases uniform corrosion and is inversely proportional to corrosion resistance (R_P_), which decreases with increased I_CORR_. This is why the value of R_P_ recorded in Table 2 was the highest for SLA and the lowest for ANO. The values of the parameters presented in Table 2 thus confirm that the uniform corrosion of the implant systems decreased in the order of SLA < MIX < ANO.

It can be noted from CCT curves (Figure 4) that the current values for all tested groups increased in the first few seconds of stepping up of the constant potential. Finally, SLA showed the lowest current whereas ANO recorded the highest values, indicating a higher dissolution rate of the surface film of the implant system and also the occurrence of pitting corrosion. This was indicated by the increased absolute values of current and the appearance of few current fluctuations. Further increasing the time of the experiment led to decreasing current values, which may have resulted from the blockage of the surface pits. The intensity of pitting corrosion here was weak, because the initiated pits did not propagate but were blocked as indicated by the decrease of currents with time. The current measured for MIX showed behavior similar to that obtained for ANO but with less absolute value and more fluctuations, indicating a higher tendency for pitting corrosion compared with ANO. The current‐time behavior thus indicates that SLA had the highest corrosion resistance, whereas ANO had the lowest and MIX was between them. Therefore, the uniform corrosion of these groups increased in the order SLA < MIX < ANO.

From the electrochemical data from all the above‐mentioned techniques, it should be concluded that SLA showed higher corrosion resistance compared with ANO. In contrast, the SLA group showed less resistance to pitting corrosion and inferior behavior in repassivation if the film oxide was broken, indicating that SLA showed increased resistance to uniform corrosion but less resistance if pitting corrosion was initiated. In all cases, MIX showed intermediate behavior.

The results of this study support that the electrochemical properties of joints between Co–Cr overdentures and Ti implants are dependent on the electrochemical properties of the treated implant surface. Given that modern surface‐roughening techniques significantly change the electrochemical properties of Ti implants (Al Jabbari et al., 2016), further research should be conducted to identify the best combinations of Co–Cr alloys and Ti implants that should be used with implant‐retained superstructures. This might decrease susceptibility to intraoral corrosion, extending the service lifetime of implants and prosthetic components. Only two implants and one combination were tested in this study and in an almost neutral solution, but further research should be done including implants with other surface modifications (double etching, plasma spray, smoothly machined, etc.) and more aggressive solutions to simulate the environment under inflammatory situations (Messer et al., 2009; Messer et al., 2010b).

## CONFLICT OF INTEREST

No conflict of interest.

## AUTHOR CONTRIBUTIONS

Dr. Alaa Al Otaibi is the principal investigator, prepared the samples, collected and analyzed the data, and write the initial draft of the paper. Professor El‐Sayed helped in conducting the research by preparing the samples, analyzing the data, and involved in writing the experimental procedures and results sections. Mohammed Al Rifaiy performed data collection and analyzing data and reviewing the final manuscript. Dr. Zinelis helped in interpreting the data and the writing and reviewing the whole manuscript. Professor Al Jabbari provided the funding and designed the study and overall controlling the experimental procedure and was heavily involved in writing the whole manuscript with the PI and reviewing the final version and submitting the research work.
